# The new Childhood Arthritis and Rheumatology Research Alliance (CARRA) registry: design, rationale, and characteristics of patients enrolled in the first 12 months

**DOI:** 10.1186/s12969-017-0160-6

**Published:** 2017-04-17

**Authors:** Timothy Beukelman, Yukiko Kimura, Norman T. Ilowite, Kelly Mieszkalski, Marc D. Natter, Grendel Burrell, Brian Best, Jason Jones, Laura E. Schanberg, For the CARRA Registry Investigators, Leslie Abramson, Shoghik Akoghlanian, Edwin Anderson, Margaret Andrew, Kevin Baszis, Mara Becker, Heather Bell-Brunson, Heather Benham, James Birmingham, Peter Blier, Hermine Brunner, Elizabeth Chalom, Johanna Chang, Paula Charpentier, Nazma Chowdhury, Joni Dean, Fatma Dedeoglu, Marija Dionizovik-Dimanovski, Brian Feldman, Polly Ferguson, Marie Fox, Kimberly Francis, Lourdes Franco, Mary Gervasini, Ingrid Goh, Donald Goldsmith, T. Brent Graham, Thomas Griffin, Dawn Helfrich, Kristin Hickey, Mark Hoeltzel, Sarah Holtschlag, Joyce Hsu, Adam Huber, Anna Huttenlocher, Lisa Imundo, Christi Inman, Jane Jaquith, Rita Jerath, Suzy Jones, Philip Kahn, Daniel Kingsbury, Kristin Klein, Marisa Klein-Gitelman, Sara Kramer, Ann Kunkel, Sivia Lapidus, Deborah Latham, Thomas Lehman, Carol Lindsley, Sean Linehan, Jennifer Lorenzo, Bipin Malla, Alexandra Martyniuk, Thomas Mason, Karen McConnell, Deborah McCurdy, Kieran McKibben, Chivon McMullen-Jackson, Diana Milojevic, Katie Mims, Christine Moniz, Sheri Morgan, Elizabeth Murray, Karen Nicely, Kathleen O’Neil, Karen Onel, Jordan Orange, Lori Ponder, Sampath Prahalad, Marilynn Punaro, C. Egla Rabinovich, Amy Rakestraw, Susan Rauch, Luke Reichley, Ariel Rhea, Sarah Ringold, Mary Ellen Riordan, Staci Roberson, Angela Robinson, Margalit Rosenkranz, Krista Ross, Deborah Rothman, Yonit Ruas, Natasha Ruth, Rosemary Sanders, Kenneth Schikler, Nora Singer, Chelsey Smith, Heidi Stapp, Shameka Swann, Reema Syed, Ashlee Tangarone, Akaluck Thatayatikom, Danielle Trejo, Jenna Tress, Richard Vehe, Emily von Scheven, Allen Watts, Jennifer Weiss, Pamela Weiss, Jennifer Woo, Ali Yalcindag, Andrew Zeft, Lawrence Zemel, Aihua Zhu

**Affiliations:** 1 Division of Pediatric Rheumatology, The University of Alabama at Birmingham, 1600 7th Avenue South, CPP 210, Birmingham, AL 35233-1711 USA; 2 Hackensack University Medical Center, Hackensack, USA; 3 Children’s Hospital at Montefiore, Bronx, USA; 4 Childhood Arthritis and Rheumatology Research Alliance, Durham, USA; 5 Children’s Hospital Boston, Boston, USA; 6 Duke University, Durham, USA

## Abstract

**Background:**

Herein we describe the history, design, and rationale of the new Childhood Arthritis and Rheumatology Research Alliance (CARRA) Registry and present the characteristics of patients with juvenile idiopathic arthritis (JIA) enrolled in the first 12 months of operation.

**Methods:**

The CARRA Registry began prospectively collecting data in the United States and Canada in July 2015 to evaluate the safety of therapeutic agents in persons with childhood-onset rheumatic disease, initially restricted to JIA. Secondary objectives include the evaluation of disease outcomes and their associations with medication use and other factors. Data are collected every 6 months and include clinical assessments, detailed medication use, patient-reported outcomes, and safety events. Follow-up is planned for at least 10 years for each participant and is facilitated by a telephone call center.

**Results:**

As of July 2016, 1192 patients with JIA were enrolled in the CARRA Registry at 49 clinical sites. At enrollment, their median age was 12.4 years old and median disease duration was 2.6 years. Owing to preferential enrollment, patients with systemic JIA (13%) and with a polyarticular course (75%) were over-represented compared to patients in typical clinical practice. Approximately 49% were currently using biologic agents and ever use of oral glucocorticoids was common (47%). The CARRA Registry provides safety surveillance data to pharmaceutical companies to satisfy their regulatory requirements, and several independently-funded sub-studies that use the Registry infrastructure are underway.

**Conclusion:**

The new CARRA Registry successfully enrolled nearly 1200 participants with JIA in the first 12 months of its operation. Sustainable funding has been secured from multiple sources. The CARRA Registry may serve as a model for the study of other uncommon diseases.

## Background

Juvenile idiopathic arthritis (JIA) is a heterogeneous collection of childhood arthritides [1]. Even though JIA is the most common pediatric rheumatologic condition with a prevalence of approximately 1 per 1000 children, the current understanding of its pathogenesis, natural history, and long-term outcomes is limited [2].

Over the last 15 years, the adoption of highly effective biologic therapeutic agents has dramatically changed the treatment and expected outcomes in JIA. Despite the widespread use of biologic agents, important safety questions remain unanswered, particularly regarding potential adverse effects that are rare or have a long latency period. Safety information about more recently approved biologic agents in children remains very limited.

Furthermore, there is little published high-quality evidence to guide pediatric rheumatologists in the management of childhood-onset systemic lupus erythematosus (cSLE), juvenile dermatomyositis (JDM), localized scleroderma, and other less common conditions.

The many current challenges in the treatment of pediatric rheumatic disease were the principal motivation for creation of the Childhood Arthritis and Rheumatology Research Alliance (CARRA) Registry. Herein, we present the history of the CARRA Registry, describe its design and rationale, and present the characteristics of the patients enrolled in the first 12 months of its operation.

## Methods

### Origins

CARRA was founded in 2002 with the mission to improve the care of children with rheumatic disease by fostering and conducting high-quality clinical and translational research. Since its founding, CARRA has grown to include 460 members in the United States and Canada, including 257 pediatric rheumatologists with sufficient fellowship training to qualify for certification by the American Board of Pediatrics or the Royal College of Physicians and Surgeons of Canada, respectively. In 2014, CARRA became legally recognized in the United States as an incorporated non-profit scientific organization under the name “CARRA Inc.” [3].

The initial CARRA Registry (now referred to as the “CARRA Legacy Registry”) was established in 2009 through funding from the National Institutes of Health [4]. This funding established the organizational, clinical research, and informatics framework for a 60-site, national registry and enabled development of a multi-center prospective observational study of children with a wide variety of defined rheumatic conditions. During its operation from 2010 through 2014, the CARRA Legacy Registry successfully enrolled the largest number of prospectively followed pediatric rheumatology patients to date: 9,587 participants including 6,607 with JIA, 1,217 with cSLE, and 688 with JDM. Data from the Legacy Registry were analyzed and presented in several peer-reviewed publications [5–16].

To create a scalable registry infrastructure for secure data collection and sharing of research data, the Legacy Registry combined established mechanisms for web-based electronic data collection with new, innovative approaches for data sharing [17]. The Legacy Registry also supported a robust training initiative for site investigators and research coordinators, helping create infrastructure for research at Registry sites and promote an overall culture of universal participation in research.

Despite the obvious success of the CARRA Legacy Registry, there were limitations due to data collection procedures. Legacy Registry participants represented a convenience sample and the generalizability of data was difficult to assess. A parsimonious set of data elements was collected to demonstrate feasibility of the new infrastructure, and detailed medication information was not included. Owing to limited funding, the collection of every 6-month follow-up visit data was not systematic. Therefore, unbiased detailed analyses of medication safety and effectiveness were not possible.

Nevertheless, the Legacy Registry successfully demonstrated the capabilities and infrastructure of the CARRA network to enroll and collect longitudinal data on large numbers of pediatric rheumatic disease participants. This provided the foundation for a suitable platform to conduct United States Food and Drug Administration (FDA) approved, post-marketing pharmacosurveillance, as well as rigorous comparative effectiveness research. In order to fulfill the new missions and accommodate new funding sources, the Legacy Registry closed to enrollment and follow-up in October 2014. Subsequently, in July 2015, the first subjects with JIA were enrolled in the new CARRA Registry.

### Objectives

The primary objective of the CARRA Registry is to prospectively collect data essential to evaluate the safety of therapeutic agents in children, adolescents, and young adults with childhood-onset rheumatic diseases. The concept of a disease-based (rather than drug-based) registry for pharmacosurveillance gained important initial traction in May 2009, when the FDA held a public workshop on developing a consolidated pediatric rheumatology observational registry [18]. Over the subsequent years, it was increasingly recognized by all stakeholders that the historical model of drug-based individual prospective registries for each new therapeutic agent was unsustainable and inadequate owing to several challenges [19]. First, competition for enrollment of subjects between individual drug-based registries created difficulties in recruiting an adequate number of comparator patients. Second, dynamic and complex medication histories made the assignment of patients to a single registry implausible. In addition, sample sizes and duration of longitudinal follow up in the existing drug-based registries were generally too small to detect uncommon but important events.

The CARRA Registry has multiple secondary objectives, including documentation of the clinical course and drug treatment patterns of patients over time, evaluation of clinical outcomes (including patient reported outcomes) associated with the use of therapeutic agents, and the evaluation of factors other than drug treatment that are associated with specific disease outcomes. The addition of targeted biospecimen collection to Registry data collection will enable translational studies of disease pathogenesis and response to therapy.

### Clinical sites

CARRA Registry sites are comprised of pediatric rheumatology practices that contain at least one active pediatric rheumatologist member of CARRA and are located throughout the United States and Canada.

### Enrollment procedures

Potential participants are recruited from activated CARRA Registry clinical sites. The overall inclusion criteria for the CARRA Registry are simple: onset of rheumatic disease prior to age 19 years old (16 years old for JIA) and current age less than 21 years. However, resource constraints initially limited enrollment to children diagnosed with JIA. In addition, to facilitate cohort studies within the Registry database, there was preferential enrollment (produced via per-patient payments to the sites) of children newly diagnosed in the prior 6 months or newly initiated on therapy with methotrexate or a biologic agent. More recently, children with systemic JIA or with a history of arthritis in 5 or more cumulative joints were also preferentially enrolled. The enrollment of children with SLE began in March 2017, and enrollment of children with JDM and localized scleroderma and systemic sclerosis is anticipated later in 2017.

The Registry was approved by Duke University Institutional Review Board (IRB) and each participating site obtained local IRB approval. Fourteen sites use a reliant IRB model in which their local IRB has ceded review to the Duke IRB. Eligible patients are consented for participation by site investigators and research coordinators in the usual manner. The option for electronic informed consent using an interactive tablet device will begin in 2017. Registry participants agree to provide patient/parent reported outcomes at each Registry visit and to be contacted by phone should they discontinue regular medical care at a CARRA Registry site (e.g., transition to an adult rheumatology care provider). Previously collected data from former Registry participants can be linked to data in the new CARRA Registry using the site-provided CARRA Legacy Registry identification number. Participants also agree to potential linkage of their data to external data sources (e.g., cancer registries) with protection of their personal identity. The CARRA Registry maintains two databases for these purposes: one with a limited research data set available to authorized researchers, and a second, separate database containing personal identifiers and contact information, which may only be viewed by a consenting participant’s site and the ‘honest broker’ staff at the central data coordinating call center. This arrangement provides an IRB-approved buffer between data for research versus data for participant contact. Participants also have the option to consent for the future collection of biospecimens.

### Data collection

Data are collected in the context of routine clinical care but cannot currently be uploaded directly from an electronic health record. CARRA pays sites for each visit with completed data entry, and most sites employ a research coordinator. Table 1 lists the baseline data collected at the time of enrollment. These same data elements are collected or updated at subsequent follow-up visits occurring approximately every 6 months as part of routine clinical practice. Follow-up data are also collected at initiation of treatment with methotrexate or a biologic agent, irrespective of whether this occurs at a routine 6-month follow-up visit. There is significant overlap between the current Registry data elements and the Legacy Registry data elements; however, the current Registry additionally collects laboratory results, detailed information about medication use, additional clinical assessments, and more patient-reported outcomes. Precise details about data elements may be provided by CARRA to potential collaborators upon request. As shown in Table 1, multiple derived measures, including the American College of Rheumatology (ACR) Pediatric Response [20] and Disease Flare [21], the Juvenile Arthritis Disease Activity Score (JADAS) [22, 23], and the provisional ACR definition of inactive disease [24], can all be determined from data collected at each Registry visit.

**Table 1:** Data items collected in the CARRA Registry

Data element
Demographics: birthdate, race, sex, household income
Other medical diagnoses
Family medical history
Health insurance provider
Dates of onset of disease symptoms, disease diagnosis, and first pediatric rheumatology evaluation
Current and past rheumatology medications: start date, stop date, dose, frequency, route of administration, reason for discontinuation
Current glucocorticoid dose and presence of any glucocorticoid use since last visit (oral, intravenous, or intra-articular)
JIA ILAR category and associated inclusion criteria
ANA, Anti-CCP, RF, HLA-B27 results
Total number of joints ever affected by arthritis (<5 or ≥5)
Disease manifestations in the past 2 weeks: fever, rash, generalized lymphadenopathy, hepatomegaly, splenomegaly, serositis, psoriasis
Height, weight, blood pressure
Total number of joints with active arthritis
Total number of joints with limited range of motion
Presence and number of tender entheses
Presence of clinically active sacroiliitis
Duration of morning stiffness
Modified Schober test measurement
Radiographic damage
Imaging evidence of sacroiliac joint damage
Imaging evidence of active sacroiliitis
Presence of uveitis (ever)
If uveitis ever present: date of diagnosis, date of most recent eye examination, current best corrected vision, ever/current use of steroid eye drops, ever use of intra-ocular or sub-tenon glucocorticoid injections, presence of anterior chamber cells, uveitis complications, eye surgery
Laboratory results (if obtained): AST, ALT, C-RP, ESR, ferritin, WBC, % neutrophils, hemoglobin, platelet count, total cholesterol, triglycerides
Physician global assessment of disease activity
Study subject contact information
Patient/parent global assessment of overall well-being, patient/parent assessment of disease activity
Childhood Health Assessment Questionnaire (CHAQ)
Pain intensity, PROMIS® pain interference, and pain due to rheumatic condition
PROMIS® upper extremity physical function, PROMIS® mobility
PROMIS® Pediatric Global Health 7

*JIA* juvenile idiopathic arthritis, *ILAR* International League Against Rheumatism, *ANA* anti-nuclear antibody, *CCP* cyclic citrullinated peptide, *RF* rheumatoid factor, *AST* aspartate aminotransferase, *ALT* alanine aminotransferase, *CR-P* c-reactive peptide, *ESR* erythrocyte sedimentation rate, *WBC* white blood cell count, *PROMIS* Patient Reported Outcomes Measurement Information System

In addition to the standard clinical measures and history collected at each follow-up, the CARRA Registry systematically collects data about safety events, specifically serious adverse events (SAEs) and events of special interest (ESIs). SAEs are defined by the standard criteria used by the FDA and others [25]. ESIs are protocol-defined, pre-specified events of particular concern and interest because of a possible association with newer therapeutic agents, but that might not meet the definition of a SAE. Examples of ESIs include optic neuritis and tuberculosis. Data about safety events are reported as soon as they are discovered by CARRA Registry site investigators, irrespective of the time elapsed since the last data entry. Data are entered using a standard form that includes detailed information about the event onset, diagnostic studies, event treatment and outcome, and recent medications. MedDRA codes are assigned to all SAE and ESI centrally at the data coordinating center by clinical coding specialists. Additional documentation of safety events, such as hospital discharge summaries or pathology reports, are obtained to increase the specificity and accuracy of any rare safety events identified.

### Patient reported outcomes

There is increasing recognition of the importance of patient reported outcomes (PROs) as valuable study endpoints. As shown in Table 1, the CARRA Registry collects patient and/or parent-proxy disease assessments, as well as relevant PROMIS® measures, such as the Pediatric Global Health (PGH-7) Measure. Electronic collection of PROs has been initiated at Registry sites in 2017 using tablet devices, with the option for research participants to also submit PROs electronically between clinical encounters using their own mobile device at home.

As a member organization of the PCORI-funded ‘Patients, Advocates and Rheumatology Teams Network for Research and Service’ (PARTNERS) Consortium Patient-Powered Research Network [26], the CARRA Registry supports re-contact of Registry participants for collection of new and/or follow-up PROs. PARTNERS also uses the Registry informatics infrastructure to house PRO and associated phenotypic data contributed from other PARTNERS organizations, thereby making this data available for simultaneous, federated querying across all contributed data sources.

### Data entry and validation

The Registry data coordinating center is the Duke Clinical Research Institute (DCRI). DCRI personnel have trained all clinical sites on appropriate practices for data collection following standard operating procedures. Data are entered using a web-based interface with programmed validity and consistency checks. If incomplete or inaccurate data are subsequently identified, data clarification requests are sent to the sites until the issues are resolved and all required data are complete. In order to ensure full compliance with FDA regulations for acceptable use of electronic records for clinical trials (i.e., 21 CFR Part 11 compliance), detailed audit trails are maintained for all relevant entries or subsequent revisions of data.

### Long-term follow-up

The CARRA Registry intends to collect data for a minimum of 10 years for each enrolled patient. Participants continue to contribute data at a CARRA Registry site as long as they continue to receive care there. If enrolled patients discontinue care from a Registry site (e.g., because of geographic relocation or aging out of pediatric care) their subsequent follow-up is transferred to a centralized Registry call center at the DCRI to maximize the possibility of maintaining long-term contact and longitudinal data collection for each participant. The telephone call center individually contacts each participant and continues to collect patient-reported data about medication use, disease activity, PROs, and safety events. This approach has been successful in other large long-term observational studies performed by the DCRI [27, 28].

### Pharmacosurveillance studies

CARRA Inc., as sponsor of the CARRA Registry, has established agreements with pharmaceutical companies to assemble clinical datasets powered to detect pharmacosurveillance safety signals, while also satisfying post-marketing commitments and requirements to regulatory authorities, such as the FDA. Currently, CARRA has active agreements to study the safety of canakinumab (Novartis Pharma AG, Basel, Switzerland) in children with systemic JIA and tocilizumab (Roche Pharmaceuticals, Basel, Switzerland) in children with polyarticular-course JIA. CARRA maintains ownership of all Registry data, as well as full scientific independence and right to publish.

### Sub-studies

One of the organizing principals for the CARRA Registry has been the capability to easily add new, modular sub-studies layered upon existing registry infrastructure. With funding from NIAMS/NIH [29], multiple disease-specific Consensus Treatment Plans (CTPs) were developed, standardized, and published contemporaneously with the inception of the CARRA Legacy Registry [30–34]. Following the success of the Arthritis Foundation-funded pilot study of the systemic JIA CTPs [35], funding was obtained from Genentech to conduct a full-scale study entitled “First-line Options for Systemic JIA Treatment (FROST)” and enrollment began in 2016. A similarly designed study of the polyarticular JIA CTPs, entitled “Start Time Optimization in Polyarticular JIA (STOP-JIA)” and funded by PCORI is currently enrolling participants. Companion biospecimen collection for translational studies has been independently funded for both studies: STOP-JIA by the Arthritis Foundation and CARRA; and FROST by the Systemic JIA Foundation. Separate from the CTP-based studies, a modular study to pilot return of research results to patients has completed (NIH/NLM) [36], and a new study to validate PROs among children with JIA and childhood-onset SLE (NIH/NIAMS) [37] is in progress. These studies represent the first of many such modular observational sub-studies anticipated for the CARRA Registry.

### Interventional studies

Interventional studies leveraging the CARRA Registry infrastructure and using the CARRA Registry for data collection are under development. Both explanatory/efficacy studies (e.g., placebo-controlled, randomized clinical trial) and pragmatic/effectiveness studies (e.g., large cluster-randomized study of two standards of care) could be incorporated. In addition to the efficiencies provided by the Registry infrastructure, there are other potential benefits of conducting interventional studies of a subset of patients enrolled in the Registry. The disease course and outcomes of patients who meet the interventional study’s inclusion criteria but who are not enrolled in the sub-study can be observed in the Registry, thus providing insight into the external validity and generalizability of the interventional study results. Also, interventional study subjects will remain in the Registry following the conclusion of the study, thus greatly aiding the assessment of the long-term effects of the intervention.

### Biospecimen collection

Biospecimens initially will be collected from patients enrolled in funded sub-studies to investigate specific translational study aims; plasma, serum, whole blood, and RNA are currently being collected for the STOP-JIA and FROST CTP studies. Future goals include the collection of biospecimens from all Registry participants and the creation of a CARRA biorepository to make the samples available to investigators.

### Data sharing

The CARRA Registry utilizes a data infrastructure that provides each participating site with full access to all data its investigators contribute to the network, as well as the ability for each Registry investigator to perform de-identified, aggregate queries of data housed across all network sites [17]. In addition, data access is provided in a tiered fashion, enabling authorized investigators to query for results across multiple CARRA studies and data sources over time, while providing filtering of patient identifiers according to investigator permissions. Specific queries can return comprehensive, longitudinal data on individuals participating in different studies.

CARRA has formulated standardized policies for data and biospecimen sharing (https://carragroup.org/policies_templates) and launched an online platform for investigators to apply for and track the progress of their data and biospecimen sharing requests. Existing data sharing policies are designed for broad access to data while providing formal mechanisms encouraging collaboration rather than competition by investigators, ensuring opportunities and equitable treatment for experienced investigators, junior faculty, and fellows to pursue their research interests, and prioritizing data access to active contributors to the Registry, regardless of site size. Access to data and biosamples is not restricted to CARRA Registry sites.

### Financial support

The CARRA Registry is owned and supported by CARRA Inc. The Arthritis Foundation financially supports CARRA Inc., including significant funding for the operation of the CARRA Registry. CARRA Inc. receives funds from pharmaceutical companies in exchange for access to pre-defined sets of post-marketing safety surveillance data. Independent funding for other Registry studies and sub-studies helps to support the general infrastructure costs of the Registry.

### Leadership

The CARRA Registry is overseen by an Executive Committee (Chair, Vice-Chair, Scientific Director, CARRA Manager of Research Operations, and DCRI project lead) who receive salary support from CARRA. To develop expertise in the operational and scientific aspects of the Registry and facilitate succession planning, CARRA sponsors year-long Registry research internships for pediatric rheumatologists who have completed clinical training.

## Results

As of July 2016, there were 49 active CARRA Registry sites that had enrolled at least one patient. Sites in the United States were broadly representative of the distribution of pediatric rheumatology centers and included at least 1 center from each of the 9 U.S. census divisions.

**Fig. 1:**
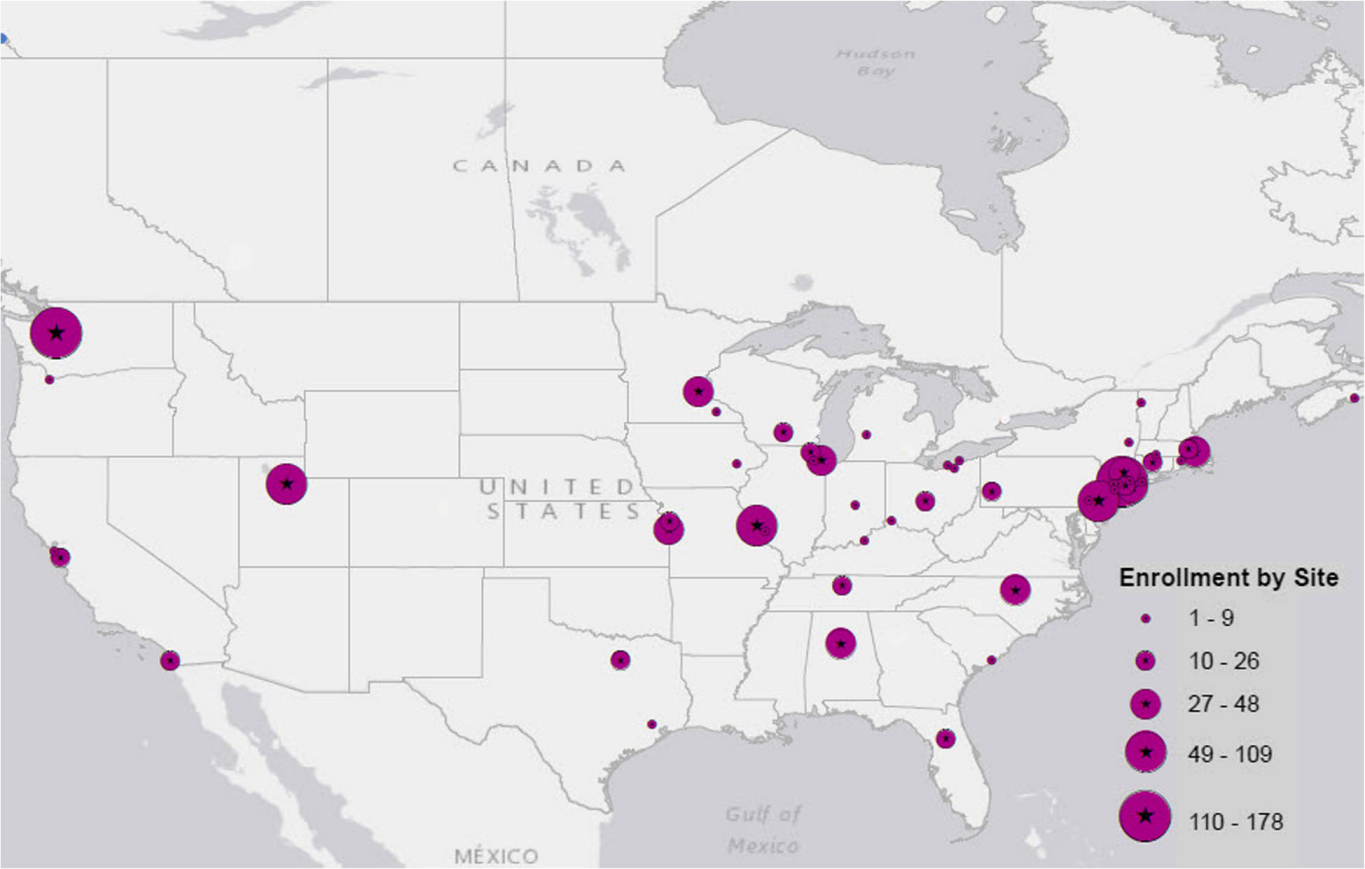
Location of CARRA Registry participating clinical sites and the number of patients enrolled in the first 12 months. The size of the circle corresponds to the number of patients enrolled, as shown in the figure legend

As of July 2016, 1,192 patients with JIA were enrolled in the CARRA Registry and had completed data entry with less than 1% missing data. The median number of patients with completed data entry at each Registry site was 14, and the largest number at a single site was 178. Thirteen sites had completed data entry for 30 or more participants. The enrollment totals by site location are shown in the Fig. 1.

The participants’ characteristics at the time of enrollment are shown in Table 2. Owing to preferential enrollment, patients with a history of polyarthritis (75%) and systemic JIA (13%) were over-represented compared to the general population of children with JIA. Most participants were relatively early in their disease course, with median disease duration of 2.6 years (interquartile range (IQR) 0.5–6.2). Many patients had active disease at the time of enrollment and the median physician global assessment of disease activity was 1.5 out of 10 (IQR 0 – 3.0).

**Table 2:** Characteristics of the patients enrolled in the CARRA Registry in the first 12 months of operation. All values are taken from the enrollment visit. Columns 3–5 are not mutually exclusive and patients may appear more than once

	Frequency (%) or Median (25–75%)			
Characteristic	All Participants (*N* = 1,192)	Recent Diagnosis^a^ (*N* = 295)	New MTX Use^b^ (*N* = 141)	New Biologic Use^b^ (*N* = 147)
Age at enrollment (years)	12.4 (7.9–15.7)	10.2 (4.8–14.1)	11.5 (6.8–14.3)	12.7 (7.2–16)
Female	887 (74%)	201 (68%)	109 (77%)	105 (71%)
Race/Ethnicity:				
White	962 (81%)	240 (81%)	112 (79%)	115 (78%)
Black/African-American	58 (5%)	15 (5%)	5 (4%)	8 (5%)
Asian	48 (4%)	15 (5%)	8 (6%)	4 (3%)
Hispanic/Latino	135 (11%)	30 (10%)	14 (10%)	20 (14%)
Middle Eastern/North African	5 (0.4%)	3 (1%)	1 (0.7%)	1 (0.7%)
Native American, American Indian, Alaskan Native	15 (1%)	2 (0.7%)	1 (0.7%)	2 (1%)
Native Hawaiian or Other Pacific Islander	12 (1%)	4 (1%)	2 (1%)	1 (0.7%)
Private health insurance	890 (75%)	217 (74%)	101 (72%)	112 (76%)
Disease duration (years)	2.6 (0.5–6.2)	0.1 (0–0.3)	1.1 (0–4)	2.2 (0.5–6.1)
ILAR category:				
Oligoarthritis, persistent	152 (13%)	90 (31%)	28 (20%)	16 (11%)
Oligoarthritis, extended	102 (9%)	6 (2%)	5 (4%)	11 (7%)
Polyarthritis, RF-	510 (43%)	88 (30%)	66 (47%)	62 (42%)
Polyarthritis, RF+	101 (8%)	18 (6%)	14 (10%)	14 (10%)
Psoriatic arthritis	57 (5%)	20 (7%)	9 (6%)	9 (6%)
Enthesitis related arthritis	104 (9%)	44 (15%)	11 (8%)	20 (14%)
Systemic arthritis	154 (13%)	26 (9%)	6 (4%)	13 (9%)
Undifferentiated arthritis	12 (1%)	3 (1%)	2 (1%)	2 (1%)
ANA+	457/1041 (38%)	107/255 (36%)	64/128 (45%)	64/134 (44%)
RF+	111/907 (9%)	24/213 (8%)	17/107 (12%)	15/118 (10%)
Anti-CCP+	91/585 (8%)	20/129 (7%)	17/74 (12%)	14/73 (10%)
HLA-B27+	98/608 (8%)	32/179 (11%)	15/75 (11%)	16/83 (11%)
Polyarthritis course	895 (75%)	151 (51%)	100 (71%)	107 (73%)
Uveitis, ever	94 (8%)	3 (1%)	8 (6%)	19 (13%)
Number of active joints	1 (0–3)	2 (1–6)	3 (1–6)	3 (1–5.75)
Physician global assessment	1.5 (0–3.0)	3 (1.5–5)	3 (1.75–4.25)	3 (2–5)
CHAQ score	0.125 (0–0.625)	0.375 (0–0.875)	0.25 (0–1)	0.5 (0–1)
ESR	8 (5–17)	9 (5–28)	9 (6–23)	9 (5–27)
CRP	0.6 (0.2–1.7)	0.8 (0.3–1.7)	1.0 (0.5–3.0)	0.8 (0.5–2.6)

Sixty-three participants were recently diagnosed and started methotrexate on the day of enrollment. Thirty-seven participants were recently diagnosed and started a biologic agent on the day of enrollment. Eleven participants were recently diagnosed and started both methotrexate and a biologic agent on the day of enrollment. Twenty-three participants total newly started both methotrexate and a biologic agent on the day of enrollment. Participants could report more than 1 race/ethnicity

*ILAR* International League Against Rheumatism, *RF* rheumatoid factor, *ANA* antinuclear antibody, *CCP* cyclic citrullinated peptide, *CHAQ* Childhood Health Assessment Questionnaire, *ESR* erythrocyte sedimentation rate, *CRP* C-reactive protein

^a^Within 6 months of diagnosis

^b^New medication was prescribed on the day of enrollment in the Registry

With respect to the creation of analytic cohorts, 295 patients were enrolled within 6 months of their diagnosis of JIA, and 265 patients were enrolled at the time of newly starting methotrexate or a biologic agent (or both). Eighty-nine patients were both enrolled within 6 months of diagnosis and also began methotrexate or a biologic agent at the time of enrollment. The characteristics of these patients are shown in Table 2. In addition, there were 50 instances of data collection at the time that established participants newly started methotrexate or a biologic agent following their enrollment.

Table 3 shows participants’ current and ever medication use at the time of enrollment. All medications used by at least 1% of participants are shown. As anticipated, participants were enriched for use of biologic agents and there was a broad variety of biologic agents used in addition to the tumor necrosis factor inhibitors. Use of oral glucocorticoids was common.

**Table 3:** Medication use at enrollment by the patients enrolled in the CARRA Registry in the first 12 months of operation

Medication	Number (%) of Current Users	Number (%) of Ever Users
Methotrexate	556 (47%)	868 (73%)
Leflunomide	52 (4%)	71 (6%)
Sulfasalazine	20 (2%)	49 (4%)
Hydroxychloroquine	49 (4%)	69 (6%)
Adalimumab	140 (12%)	235 (20%)
Etanercept	273 (23%)	505 (42%)
Golimumab	7 (1%)	13 (1%)
Infliximab	44 (4%)	95 (8%)
Any TNFi	465 (39%)	664 (56%)
Any TNFi + Methotrexate	255 (21%)	n/a
Abatacept	17 (1%)	55 (5%)
Tocilizumab	59 (5%)	115 (10%)
Tocilizumab + Methotrexate	42 (4%)	n/a
Anakinra	24 (2%)	75 (6%)
Canakinumab	29 (2%)	46 (4%)
Any IL-1 inhibitor + Methotrexate	18 (2%)	n/a
Rituximab	3 (<1%)	16 (1%)
Oral glucocorticoids	124 (10%)	560 (47%)

*TNFi* tumor necrosis factor inhibitor, *IL-1* interleukin 1, *n/a* not assessed

As of July 2016, 12 safety events had been reported including 8 serious adverse events and 3 episodes of macrophage activation syndrome. There were no deaths or incident malignancies reported.

## Discussion

The newly initiated CARRA Registry has built upon the successes of the former CARRA Legacy Registry by leveraging site investigator experience and training as well as coordinating center expertise and innovative technology for integrating and sharing data. Nearly 1,200 patients with JIA were enrolled in the CARRA Registry in the first year since its inception while the number of participating clinical sites was still accumulating. The CARRA Registry has successfully entered collaborations with two pharmaceutical companies to provide data necessary for post-marketing safety surveillance studies. CARRA investigators have successfully secured independent funding to perform observational studies of the published CARRA CTPs. Many additional observational and interventional Registry studies and sub-studies, as well as translational studies using Registry-collected biospecimens linked to Registry clinical data, are anticipated in the near future and will benefit greatly from the growing Registry infrastructure.

Registry data to date demonstrate the ability to rapidly enroll patients with clinical data of the highest utility, namely those newly diagnosed (295; 25% of overall enrollees) and newly starting methotrexate or biologic agents (265; 22% of overall enrollees). Newly diagnosed patients provide prospective data about the natural history of disease and its treatment course that are less prone to bias; nevertheless, the CARRA Registry is not truly population-based and data may not be generalizable to all children diagnosed with JIA. Patients newly starting medications establish retrospective and prospective analytic cohorts within the Registry, enabling the unbiased evaluation of the safety and effectiveness of therapeutic agents. This “new user” analytic design greatly improves the validity of study results [38, 39].

One of the keys to the sustained success of the CARRA Registry will be providing investigators as well as participants with added value for their engagement in the Registry. The most obvious added value will result from published studies and improved knowledge of the safety and effectiveness of therapeutic agents. For research investigators, the ability to freely access and analyze data collected on their own patients, in combination with straightforward policies and rapid processes for previewing and then accessing data sourced across the entire network, will foster additional opportunities to conduct research of interest to them in a timely fashion. For clinicians, providing personalized, up-to-date benchmarks of their own site’s practices and therapeutic decisions in context to many other pediatric rheumatologists is likely to be of considerable interest and benefit, and should catalyze meaningful follow-up discussions and analyses, supporting “learning health system” cycles [40]. For patients and their families, providing feedback of results of self-identified importance, and incorporating patient preferences across the spectrum of research into long-term outcomes, is likely to be of high value.

## Conclusion

Single product registries in the United States have disappointed both patient and provider communities due to their limited enrollment and inability to address many important questions. The disease-specific CARRA Registry offers an alternative approach designed to directly address these concerns and provide broader opportunities to streamline clinical research in understudied populations. We hope the CARRA Registry will serve as a valuable paradigm for the study of uncommon diseases.

## Abbreviations

ACR: American College of Rheumatology
CARRA: Childhood arthritis and rheumatology research alliance
cSLE: Childhood-onset systemic lupus erythematosus
CTPs: Consensus treatment plans
DCRI: Duke Clinical Research Institute
ESI: Event of special interest
FDA: United States Food and Drug Administration
FROST: First-line options for systemic JIA treatment
IRB: Institutional review board
JADAS: Juvenile arthritis disease activity score
JDM: Juvenile dermatomyositis
JIA: Juvenile idiopathic arthritis
PARTNERS: Patients, Advocates, and Rheumatology Teams Network for Research and Service
PGH-7: Pediatric Global Health 7
PROs: Patient reported outcomes
SAE: Serious adverse event
STOP-JIA: Start time optimization in polyarticular JIA
